# Anterior total hip arthroplasty using a metaphyseal bone-sparing stem: component alignment and early complications

**DOI:** 10.1186/s13018-016-0383-0

**Published:** 2016-04-22

**Authors:** Mohammed M. Ahmed, Thomas J. Otto, Berton R. Moed

**Affiliations:** Department of Orthopaedic Surgery, Saint Louis University School of Medicine, 3635 Vista Ave., Desloge Towers 7th Fl, St. Louis, MO 63110-2539 USA

**Keywords:** Total hip arthroplasty, Anterior approach, Bone-preserving stem, Complications

## Abstract

**Background:**

Limited-incision total hip arthroplasty (THA) preserves hip abductors, posterior capsule, and external rotators potentially diminishing dislocation risk. However, potential complications also exist, such as component malposition. Specific implants have been manufactured that enhance compatibility with this technique, while preserving metaphyseal bone; however, little data exists documenting early complications and component position. The purpose was to evaluate primary THA using a curved, bone-sparing stem inserted through the anterior approach with respect to component alignment and early complications.

**Methods:**

In a retrospective analysis of 108 cases, the surgical technique was outlined and the occurrence of intraoperative fractures, postoperative dislocations, infection, and limb length inequality was determined. Femoral stem and acetabular cup alignment was quantified using the initial postoperative radiographs. Patient follow-up averaged 12.9 (range 2 to 36) months.

**Results:**

There were eight (7.4 %) complications requiring revision surgery in three (2.8 %) patients with three (2.8 %) infections and three (2.8 %) dislocations. Intraoperative complications included one calcar fracture above the lesser trochanter. Leg length inequality >5 mm was present in three (2.8 %) patients. Radiographic analysis showed that femoral neutral alignment was achieved in 95 hips (88.0 %). All femoral stems demonstrated satisfactory fit and fill and no evidence of subsidence, osteolysis, or loosening. An average abduction angle of 44.8° (±5.3) and average cup anteversion of 16.2° (±4.2) were also noted.

**Conclusions:**

Although the technique with this implant and approach is promising, it does not appear to offer important advantages over standard techniques. However, the findings merit further, long-term study.

## Background

Complications after total hip arthroplasty (THA) include hip dislocation, abductor weakness, leg length inequality, and gait disturbances, any of which can require revision THA [1]. Common factors that may influence mechanical complications include the surgical approach, component alignment, and prosthetic design. Controversy exists regarding the potential influence of the specific surgical approach on outcomes [2–4].

The direct anterior approach (DAA) for THA is a surgical technique that preserves the hip abductors, the posterior capsule, and the short external rotators [1, 5, 6]. In this way, the need for postoperative hip precautions may be reduced. Another concept gaining recent interest is bone preservation during hip arthroplasty [7]. Subsequently, proximal metaphyseal filling femoral implants have become commercially available. Due to their short, curved shape, these implants may also facilitate femoral component positioning when using the DAA.

The Fitmore hip stem (Zimmer, Warsaw, IN) is one such prosthesis and is marketed by the manufacturer as a bone-preserving implant. To the best of our knowledge, there has been little published regarding the use of this type of implant with the DAA limited to one abstract describing the surgical technique [8]. The purpose of this study was to evaluate the DAA for primary THA using the Fitmore hip stem with respect to achieving acceptable component alignment and early complications encountered.

## Methods

**Fig. 1:**
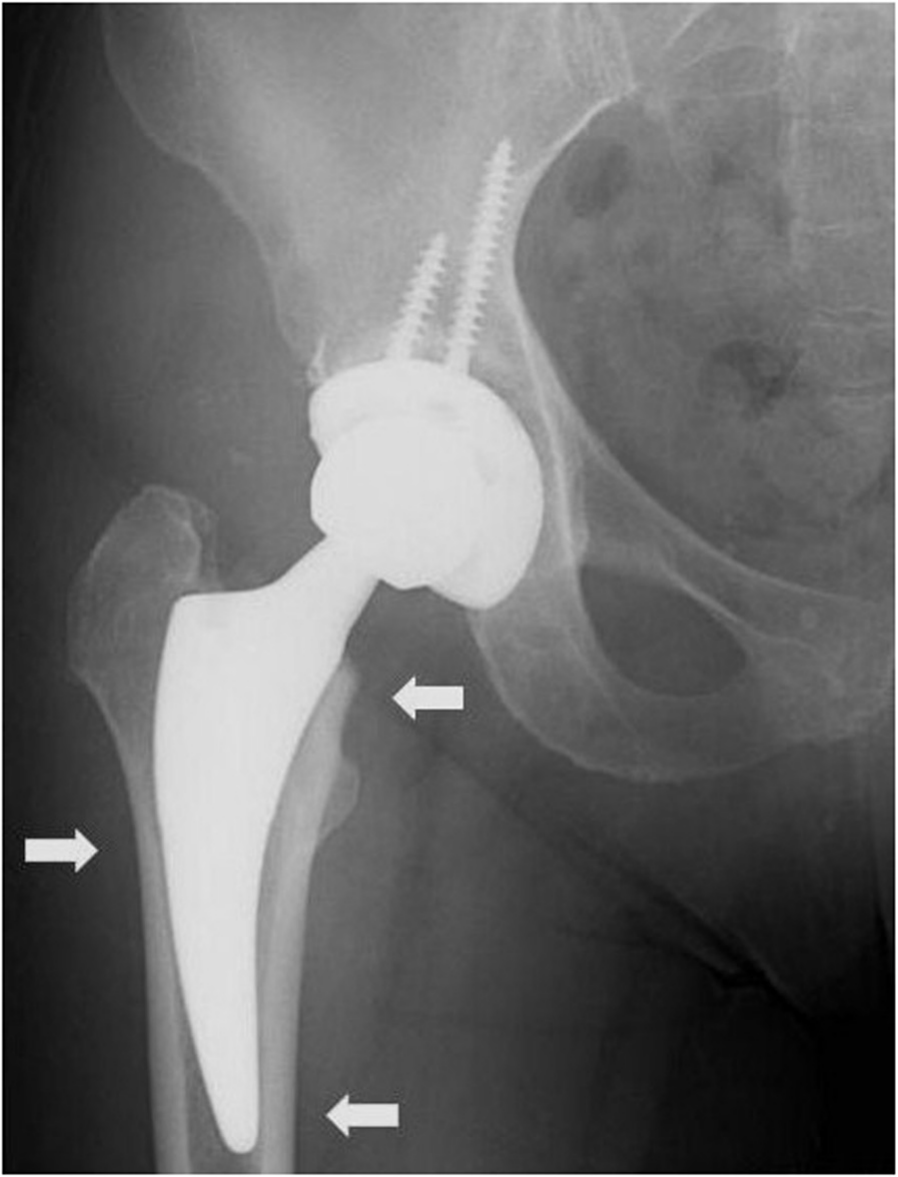
Satisfactory femoral neutral alignment was achieved if three points of bony apposition (*arrows*) were obtained at the calcar proximally, on the lateral endosteal surface in the middle, and at the medial endosteal surface with the tip of the stem distally as shown in the figure

**Fig. 2:**
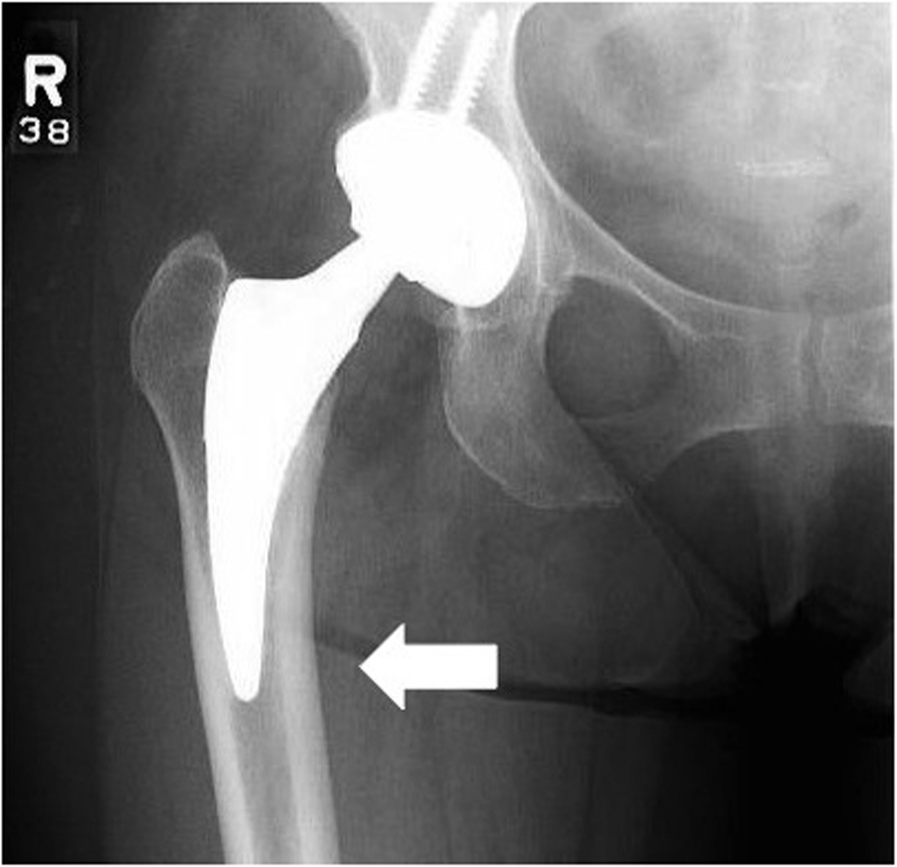
A stem placed in varus is demonstrated based on the three-point apposition method. The distal tip of the stem is noted to lack apposition with the medial endosteal surface (*arrow*)

After Institutional Review Board approval, information from all consecutive patients who underwent primary THA at our center by the senior surgeon from 2008 to 2011 was retrieved using an existing patient database. Within this period of time, a total number of 128 primary THA procedures were performed using the DAA. Exclusion criteria included cases with inadequate follow-up (<2 months) or where a different implant was used. However, review of these 20 cases that were excluded revealed no complications. A total of 108 primary THAs were identified for final review. The charts of these cases were reviewed to identify patient information including demographics, indications for surgery, and preoperative radiographs (Table 1). Indications for surgery included advanced arthritis recalcitrant to conservative measures in the setting of primary osteoarthritis, posttraumatic arthritis, rheumatoid arthritis, avascular necrosis (AVN), femoral neck nonunion, and AVN as a sequela of a failed slipped capital femoral epiphysis in one patient. The senior author uses the DAA with a short curved stem for all primary THA. While no specific indications for the DAA have clearly been defined, we feel that this approach may especially be more advantageous in larger patients, as the subcutaneous fat in the anterior thigh region may be minimal compared to other areas of the hip. Relative contraindications to the DAA using short curved stems in primary THA include patients with previous hip surgery with retained instrumentation requiring removal, patients with proximal femoral canal deformity, or complex cases where extensive surgery may be required (femoral shortening osteotomy). The surgical objectives were to restore pre-disease anatomic alignment of the hip joint. The mean patient age was 57.4 years (range 12–85 years). Digital templating software (TraumaCad, Voyant Health, Columbia, MD) was used to template all cases preoperatively. The Fitmore hip stem is designed to have apposition at the calcar and the lateral endosteal surface just distal to the level of the lesser trochanter. We planned to create three points of fixation of the stem within the intramedullary canal in order to obtain neutral stem alignment. These included apposition at the calcar proximally, on the lateral endosteal surface in the middle, and at the medial endosteal surface distally on a standard pelvis radiograph (Fig. 1). Based on our interpretation, a stem was said to be in neutral alignment if all three points of fixation were achieved. The stem was deemed to be in varus if the distal medial endosteal apposition site was not obtained (Fig. 2). Conversely, a valgus stem was one in which the stem did not achieve apposition at the calcar proximally. While the manufacturer describes these parameters specifically for the Fitmore stem, we believe that this novel radiographic assessment can be utilized for any short curved stem. Targets for acetabular implant alignment were 45° of abduction and 20° of cup anteversion. Alignment was said to be satisfactory if abduction angles were within 35°–50° and if anteversion was within 10°–25° as previously described [1, 9].

**Table 1:** Patient demographic data

Demographics	Results
% male:% female	39 %:61 %
Mean age	57.7 (±12.6)
% right:% left	50 %:50 %
Indications for surgery (*N*)	
Primary osteoarthritis	92
Posttraumatic arthritis	11
Femoral neck nonunion	2
Rheumatoid arthritis	2
AVN s/p SCFE	1
Mean preoperative Harris Hip Score	47.5 (±11.2)
Mean hospital length of stay (days)	3.6 (±.9)
Mean follow-up (months)	12.9 (±9.1)

Patient demographic data with subdivided groups both preoperative and postoperatively

### Surgical technique

After the institution of general endotracheal anesthesia, the patient is placed on a PROFx table (Mizuho OSI, Union City, CA) and secured in the supine position with both feet secured in the appropriately sized holding devices. Bilateral leg compression devices are placed. Patients receive appropriate preoperative antibiotics per the institution’s SCIP guidelines. A standard direct anterior approach total hip arthroplasty, as has been previously described, is performed [1, 5]. After the acetabular component is appropriately implanted, attention is turned to the femoral side. The operative extremity is hyper-extended, externally rotated, and adducted to facilitate proper visualization of the proximal femur. A curved chisel or curved hand rasp is used to enter the canal. The start point for entry should be cheated posteriorly and in the middle third of the osteotomy. It is important to point the tip of the curved starting chisel or hand rasp down the axis of the medullary canal. Broaching is then performed using the Fitmore system starting with the smallest size available. Visualization of the distal tip is crucial to prevent varus misalignment or medial wall cortical perforation. The most important step to ensure stability of the stem, however, is to obtain cortical contact at the level of the calcar to prevent stem subsidence. The final broach is used as the trial femoral stem and standard or extended neck configurations are attached, followed by the appropriate head size. After hip reduction, stability is assessed clinically by using the shuck test and by forceful internal and external rotation. Implant orientation, appropriate fill, offset, and leg length measurements are documented with image intensification. The trial femoral components are dislocated, removed, and replaced with the corresponding final implants.

Postoperatively, all patients are made weight bearing as tolerated unless an intraoperative fracture has occurred (one patient), in which case protective weight bearing is administered for a short time period. No postoperative hip precautions are used.

**Fig. 3:**
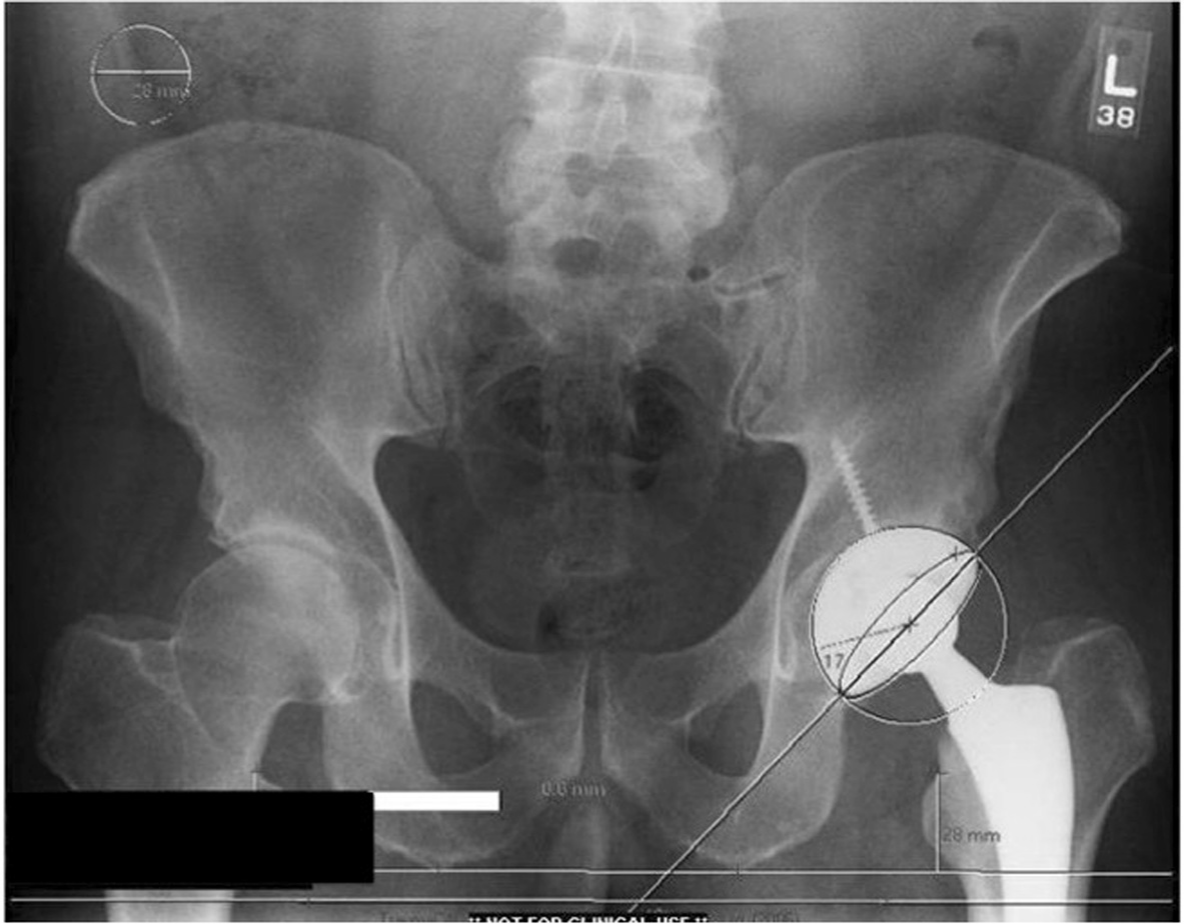
Component alignment measurements made in the same patient as shown in Fig. 2. The templating software was used to measure cup anteversion (17° in this patient), cup abduction (45°), and leg length difference (0.6 mm)

Femoral component alignment was assessed based on endosteal apposition at the three sites described earlier using postoperative standing hip and pelvis radiographs obtained at the first postoperative clinic visit. Using the same templating software, measurements for leg length difference, cup abduction angle, and cup anteversion angle were also obtained (Fig. 3). Finally, early follow-up evaluation was reviewed to determine any functional deficits or complications of the procedure. The mean patient follow-up was 12.9 (range 2 to 36) months. Descriptive statistics were used to analyze various measurements using the SPSS software (SPSS software, version 19; SPSS, Chicago, IL).

## Results

Complications were seen in eight total cases (7.4 %) of which three (2.8 %) required revision surgery (Table 2). There were three (2.8 %) postoperative infections, of which one required revision, and three (2.8 %) hip dislocations, two requiring revisions. Intraoperative complications included one calcar fracture above the level of the lesser trochanter during insertion of the femoral stem. One patient experienced a myocardial infarction perioperatively; there were no cases of deep venous thrombosis or pulmonary embolism.

**Table 2:** Complications

Complications	Number (%)
Total complications	8 (7.4)
Intraoperative fracture(s)	1 (1)
Postoperative infections	3 (2.7)
Postoperative dislocations	3 (2.7)
Postoperative myocardial infarction	1 (1)
Total number of revisions	3 (2.7)

Total complications and breakdown of individual complications for all patients

Radiographic evaluation of component alignment (Table 3) revealed a postoperative leg length inequality of a magnitude greater than 5 mm in three patients (2.8 %). The mean leg length difference was 2.6 mm (±2.2). Femoral neutral alignment, measured by the ability to obtain three points of fixation, was achieved in 95 hips (88.0 %). Of the 13 stems that did not achieve neutral alignment, all showed apposition at the calcar and lateral endosteal surface and lacked apposition at the medial endosteal surface distally, indicating a varus malalignment. All femoral stems demonstrated satisfactory fit and fill and showed no evidence of subsidence, osteolysis, or loosening at follow-up. The target ranges for acetabular cup abduction and anteversion angles were achieved in 90 patients (83.3 %) and 101 patients (93.5 %), respectively. The mean abduction angle was 44.8° (±5.3), and the mean cup anteversion was 16.2° (±4.2).

**Table 3:** Component alignment

Alignment measurements	Totals (%)	Means (range)
Postoperative leg length inequality >5 mm	3 (2.7)	
Mean leg length inequality		2.6 mm (±2.2)
Femoral neutral alignment (three-point fixation)	95 (88)	
Femoral satisfactory fit and fill	108 (100)	
Within the target cup abduction angle (30°–50°)	91 (84.3)	
Mean cup abduction angle		44.8 (±5.3)
Within the target cup anteversion angle (10°–25°)	101 (93.5)	
Mean cup anteversion angle		16.2 (±4.2)

Radiographically measured various component alignment measurements for all patients

## Discussion

Traditional approaches to THA often necessitate gluteus muscle stripping, which lead to muscle weakness, postoperative limp, and increased rehabilitation time [2–4]. The posterior approach continues to be popular because it preserves the hip abductors but still carries with it potential drawbacks [10, 11]. The anterior approach allows access through an existing intermuscular plane, theoretically decreasing some of these risks, but it also is not fault proof [1, 5, 6, 12, 13]. Potential complications of component malposition and its correlation with increased hip instability have been studied extensively [8, 14–16].

To our knowledge, there have been no reports of radiographic results or early complications of a short, curved stem using the anterior approach, serving as the impetus for this study. Furthermore, because of its curved design, standard femoral stem measurements cannot be used to determine alignment. As such, we propose a novel method to assess femoral stem alignment based on our early clinical experience. In an effort to maximize stability, we attempted to recreate three points of fixation of the stem within the intramedullary canal, two of which were medial at the proximal and distal appositional ends of the stem and laterally in the center (Fig. 1). If a stem attained apposition at all three points, it was considered to be in neutral alignment. If there was no apposition on the medial endosteal surface distally, the stem was said to be in varus (Fig. 2). In our study, radiographic analysis demonstrated 95 stems to be in neutral alignment (88 %) with the rest being in varus. Though our follow-up is limited, none of these patients showed symptoms at the latest follow-up and none demonstrated component subsidence, osteolysis, or loosening.

The overall incidence of dislocation in the setting of primary THA varies widely, ranging from 2 to 5 % [10, 11, 14, 15, 17]. Dislocation rates with the direct anterior approaches for THA also vary, ranging from <1 to 3 % [1, 6, 12, 13, 18]. In this study, there were three total anterior dislocations (2.7 %). Two of these patients required revision surgery with placement of a larger femoral head, while the last was successfully managed with a closed reduction and a brace. Of note, at the time of revision surgery, the two cases demonstrated appropriate component alignment both on the acetabular and femoral side. In this study, the mean abduction angle was 44.8° (±5.3) and the mean cup anteversion was 16.2° (±4.2) with 83.3 and 93.5 % landing within their respective target zones. This is on par with other studies in the past [1, 9]. It is uncertain what ultimately caused instability in these patients, but all three cases had a 28-mm-sized femoral head. Studies have indicated increase in dislocation rates with lower diameter femoral heads [19, 20]. This was at least partly responsible for the dislocations as increasing the size to 32 and 40 mm treated them successfully. Also, it is important to note that this is a study of the first group of patients to undergo this specific technique. Multiple studies have documented a learning curve using this approach and have reported on a decreasing number of complications as the total number of cases increases [9, 18]. Furthermore, one patient had a history of an acetabular fracture requiring open reduction and internal fixation. Inferior results in these patients have previously been documented [17, 21]. In fact, Woo and Morrey suggested that a history of hip surgery doubled the incidence of dislocation to 4.8 % when compared to no prior surgery [17].

The overall risk of infection in primary THA has been reported to be between 1 and 2 % [22, 23]. With the anterior approach specifically, the rate seems to be similar [12, 13, 18, 22]. In our study, there were three total infections (2.7 %). Two patients were successfully treated with irrigation and debridement with retention of the implants while the third required explant and a staged revision. Of note, the same sized femoral implant was utilized at the time revision, demonstrating the concept of bone preservation. This third patient also had a history of an ORIF of his acetabulum. Reports indicate significantly higher rates of infection in the setting of previous acetabular and hip trauma [21, 24].

In this study, one patient sustained a calcar fracture during stem insertion. The patient was treated with a cerclage wire, and the stem was found to be stable. The patient was treated with protected weight bearing for 6 weeks and went on to have no complications. Intraoperative fractures are a known complication of this technique ranging from 1.5 to 2.5 % [1, 12, 18]. We believe the lower rate of intraoperative fracture encountered here is due to the curved design of the stem. This allows easier placement of the stem obviating the need for greater torqueing maneuvers in order to obtain adequate visualization.

The strength of this study includes its relatively large case series with the same surgical protocol and single-surgeon technique minimizing potential confounding variables. It is the first to document femoral stem alignment for short, curved stems with the use of the DAA for THA. Limitations include its retrospective analysis and relatively short follow-up. While efforts are made to standardize clinic radiographs, retrospective analysis of such images can often times have limitations due to quality and consistency of imaging. Moreover, the observational nature of study design has its own inherent shortcomings. Finally, no long-term clinical outcomes were reviewed in this study. Despite these limitations, we feel that this study does offer valuable information with regard to this technique and implant and also describes a novel method of evaluating component alignment in short, curved, bone-sparing stems. Furthermore, we believe this method of assessment can potentially aid in preoperative planning when determining the appropriate size and orientation of the stem. Because this is only a preliminary study, we cannot validate the method at the present and believe that such a study would be warranted in the future.

## Conclusions

In conclusion, early radiographic analysis of component alignment and restoration of leg lengths is promising using a short, curved stem with the DAA. Early complication rates including infection and hip dislocation are comparable to studies utilizing traditional implants. Further studies will be needed to determine the long-term outcomes of these prostheses specifically marketed for the DAA.

## Ethics approval

The institutional review board at St. John’s Mercy Medical Center approved this project according to federal regulations (IRB Reference #: 11–057).

## References

[1] Matta JM, Shahrdar C, Ferguson T (2005). Single-incision anterior approach for total hip arthroplasty on an orthopaedic table. Clin Orthop Relat Res.

[2] Baker AS, Bitounis VC (1989). Abductor function after total hip replacement. An electromyographic and clinical review. J Bone Joint Surg Br.

[3] Horwitz BR, Rockowitz NL, Goll SR (1993). A prospective randomized comparison of two surgical approaches to total hip arthroplasty. Clin Orthop Relat Res.

[4] Masonis JL, Bourne RB (2002). Surgical approach, abductor function, and total hip arthroplasty dislocation. Clin Orthop Relat Res.

[5] Oinuma K, Eingartner C, Saito Y, Shiratsuchi H (2007). Total hip arthroplasty by a minimally invasive, direct anterior approach. Oper Orthopade Traum.

[6] Siguier T, Siguier M, Brumpt B (2004). Mini-incision anterior approach does not increase dislocation rate: a study of 1037 total hip replacements. Clin Orthop Relat Res.

[7] Glassman AH, Bobyn JD, Tanzer M (2006). New femoral designs: do they influence stress shielding?. Clin Orthop Relat Res.

[8] Yerasimides JG (2010). Use of the Fitmore(R) hip stem bone-preserving system for the minimally invasive anterior-supine approach in hip replacement. Am J Orthop.

[9] Woolson ST, Mow CS, Syquia JF, Lannin JV, Schurman DJ (2004). Comparison of primary total hip replacements performed with a standard incision or a mini-incision. J Bone Joint Surg Am.

[10] Fackler CD, Poss R (1980). Dislocation in total hip arthroplasties. Clin Orthop Relat Res.

[11] Soong M, Rubash HE, Macaulay W (2004). Dislocation after total hip arthroplasty. J Am Acad Orthop Surg.

[12] Hallert O, Li Y, Brismar H, Lindgren U (2012). The direct anterior approach: initial experience of a minimally invasive technique for total hip arthroplasty. J Orthop Surg Res.

[13] Horne PH, Olson SA (2011). Direct anterior approach for total hip arthroplasty using the fracture table. Curr Rev Musculoskelet Med.

[14] Lewinnek GE, Lewis JL, Tarr R, Compere CL, Zimmerman JR (1978). Dislocations after total hip-replacement arthroplasties. J Bone Joint Surg Am.

[15] McCollum DE, Gray WJ (1990). Dislocation after total hip arthroplasty. Causes and prevention. Clin Orthop Relat Res.

[16] Morrey BF (1997). Difficult complications after hip joint replacement. Dislocation. Clin Orthop Relat Res.

[17] Woo RY, Morrey BF (1982). Dislocations after total hip arthroplasty. J Bone Joint Surg Am.

[18] Jewett BA, Collis DK (2011). High complication rate with anterior total hip arthroplasties on a fracture table. Clin Orthop Relat Res.

[19] Howie DW, Holubowycz OT, Middleton R (2012). Large femoral heads decrease the incidence of dislocation after total hip arthroplasty: a randomized controlled trial. J Bone Joint Surg Am.

[20] Peter R, Lubbeke A, Stern R, Hoffmeyer P (2011). Cup size and risk of dislocation after primary total hip arthroplasty. J Arthroplasty.

[21] Ranawat A, Zelken J, Helfet D, Buly R (2009). Total hip arthroplasty for posttraumatic arthritis after acetabular fracture. J Arthroplasty.

[22] Bauer TW, Parvizi J, Kobayashi N, Krebs V (2006). Diagnosis of periprosthetic infection. J Bone Joint Surg Am.

[23] Zimmerli W, Trampuz A, Ochsner PE (2004). Prosthetic-joint infections. N Engl J Med.

[24] Mears DC, Velyvis JH (2001). Primary total hip arthroplasty after acetabular fracture. AAOS Instr Cours Lec.

